# Diffusion- and Perfusion-Weighted Imaging in Acute Lacunar Infarction: Is There a Mismatch?

**DOI:** 10.1371/journal.pone.0077428

**Published:** 2013-10-10

**Authors:** Alex Förster, Hans Ulrich Kerl, Holger Wenz, Marc A. Brockmann, Ingo Nölte, Christoph Groden

**Affiliations:** 1 Department of Neuroradiology, Universitätsmedizin Mannheim, University of Heidelberg, Heidelberg, Germany; 2 Department of Diagnostic and Interventional Neuroradiology, University Hospital of the RWTH Aachen, Aachen, Germany; Julius-Maximilians-Universität Würzburg, Germany

## Abstract

**Purpose:**

Characterization of lacunar infarction (LI) by use of multimodal MRI including diffusion- and perfusion-weighted imaging (DWI, PWI) is difficult because of the small lesion size. Only a few studies evaluated PWI in LI and the results are inconsistent.

**Methods:**

In 16 LI patients who underwent initial MRI within 6 hours after symptom onset and follow-up MRI within 1 week demographics, clinical presentation, and MRI findings were analyzed with special emphasis on DWI and PWI findings. Time to peak maps were classified as showing a normal perfusion pattern or areas of hypoperfusion which were further categorized in mismatch (PWI>DWI), inverse mismatch (PWI<DWI), and match (PWI=DWI). Quantitative perfusion maps were generated and analyzed by use of Signal Processing in NMR-Software (SPIN).

**Results:**

Of the 16 patients (mean age 65.5±12.9 years), 14 (87.5%) were male. Clinical symptoms comprised dysarthria (50%), hemiparesis (81.3%), and hemihypaesthesia (18.8%). Intravenous thrombolysis was performed in 7 (43.8%) patients. Clinical improvement was observed in 12 patients (75 %), while 2 (12.5%) patients showed a deterioration and another 2 (12.5%) a stable course. Acute ischemic lesions (mean volume of 0.46±0.29 cm^3^) were located in the thalamus (n=8, 50%), internal capsule (n=4, 25%), corona Radiata (n=3, 18.8%) and the mesencephalon (n=1, 6.3%). Circumscribed hypoperfusion (mean volume 0.61±0.48 cm^3^) was evident in 10 (62.5%) patients. Of these, 3 patients demonstrated a match, 4 an inverse mismatch, and 3 a mismatch between DWI and PWI lesion. Mean CBF and CBV ratios were 0.65±0.28 and 0.84±0.41 respectively. Growth of DWI lesions was observed in 7 (43.8%) and reversal of DWI lesions in 3 (18.8%) patients.

**Conclusions:**

MRI allows identification of different DWI and PWI patterns in LI, including growth and reversal of ischemic lesions. Consequently, it may serve for a better characterization of this stroke subtype and support treatment decisions in daily clinical practice.

## Introduction

Lacunar infarction (LI) accounts for up to 25% of acute ischemic strokes[1] and is defined as a small subcortical ischemic lesion with a maximal diameter <15 mm located in the basal ganglia, thalamus, internal capsule, corona radiata or brainstem caused by occlusion of a single penetrating artery due to lipohyalinosis or microatheroma[2]. The clinical presentation comprises five typical so-called lacunar syndromes (pure motor stroke, pure sensory stroke, sensorimotor stroke, dysarthria-clumsy hand syndrome, and ataxic hemiparesis)[3]. For unknown reasons, a deterioration in the clinical course can be observed in many patients with LI[4]. While LI has been assumed to be associated with a rather benign prognosis for a while[1], more recent studies demonstrated a higher mortality and risk of recurrent stroke in patients with LI in the longer perspective[5]. 

While multimodal MRI including magnetic resonance angiography (MRA), and perfusion-weighted imaging (PWI) can be used to characterize the underlying vessel pathology and degree of perfusion changes in large territorial ischemic stroke in detail, its ability to demonstrate corresponding changes in acute LI are limited due to the relatively small diameter of the penetrating arteries and the small lesion size. Only a few studies investigated PWI in LI and their results are inconsistent[6-14]. Furthermore, the question whether acute LI may benefit from acute treatment with recombinant tissue plasminogen activator (rtPA) has been a matter of debate as it was hypothesized that intravenous thrombolysis would only be effective in patients with large vessel occlusion[15,16].

In this case series of patients with acute LI admitted to our hospital we aimed to describe infarction patterns on DWI and perfusion patterns on PWI in the acute stage and at follow-up in order to provide a better understanding of its pathophysiology and to indentify ischemic lesions suited for intravenous thrombolysis.

## Material and Methods

### Patients

The study was approved by the local institutional review board (Medizinische Ethikkommission II der Medizinischen Fakultät Mannheim). Patient consent was not required by our IRB for this de-identified database (Perfusion-weighted imaging in Lacunar Infarction - PILI) due to the retrospective nature of the study and the lack of patient interaction. From a prospectively maintained MRI report database (Syngo Data Manager – SDM), we identified 698 patients with suspected acute ischemic stroke who underwent a standard stroke MRI protocol including PWI (2005-2013). Among these, 55 (7.9%) had an acute LI and of these, 16 (2.3%) patients had the first MRI within 6 hours as well as follow-up MRI within 1 week after symptom onset and formed the study cohort. Follow-up MRI was performed for different reasons, including the evaluation of treatment response after intravenous thrombolysis, deterioration in the clinical course, or to rule out ischemia-associated haemorrhage. The demographic details, clinical presentation, and acute treatment were abstracted from the case records. Standardized clinical work-up included assessment of cardiovascular risk factors, extra- and transcranial Doppler-/duplex sonography, 24-hour electrocardiogram monitoring and transthoracic or transesophageal echocardiography as well as laboratory tests according to stroke unit standard requirements in Germany[17]. Patients with any other underlying stroke etiology according to the Trial of Org 10172 in Acute Stroke Treatment (TOAST)[18] stroke subtype classification were excluded from the analysis.

### MRI Studies

Magnetic resonance imaging was performed on a 1.5-T MR system (Magnetom Sonata or Avanto, Siemens Medical Systems, Erlangen, Germany) within 6 hours after onset of symptoms. A standardized protocol was used in all patients including (1) transverse, coronal and sagittal localizing sequences followed by transverse oblique contiguous images aligned with the inferior borders of the corpus callosum (applied on sequences 2 to 5); (2) T1-weighted images; (3) T2-weighted images; (4) DWI; (5) fluid attenuated inversion recovery (FLAIR) images; (5) PWI following the first pass of contrast bolus through the brain; and (6) a 3D time-of-flight MR angiography. Perfusion-weighted imaging was acquired using a gradient-echo echo planar imaging sequence (field of view 230x230 mm, acquisition matrix 128x128, number of slices 12/19, slice thickness 6/5 mm, TR 1500/1430 ms, TE 46/30 ms, duration 1:30 minutes for Magnetom Sonata/Avanto). The contrast agent gadoteric acid (Dotarem, Guerbet, Aulnay-sous-Bois, France) was bolus injected by a power injector (Spectris MR injection system, Medrad, Volkach, Germany) with a dose of 0.1 mmol/kg of body weight at a rate of 4 ml/sec. Follow-up MRI was performed within a mean of 2.6±2.2 days (range 1 to 7 days) after onset of symptoms using the same MRI protocol except for PWI which was performed in only 4 patients.

### Postprocessing

The postprocessing of the perfusion-weighted raw images was performed by a specific software, Signal Processing In NMR (SPIN, The MRI Institute for Biomedial Research, Detroit, USA) [19]. Deconvolution with singular value decomposition (SVD) was used to create quantitative maps of mean transit time (MTT), cerebral blood flow (CBF), and cerebral blood volume (CBV). The position of the arterial input function (AIF) was automatically determined by using the maximum concentration (C_max_), time to peak (TTP) and first moment MTT (fMTT). The concentration-time curve for arteries has short fMTT, short TTP and high C_max_. Twenty voxels, which best fit these properties were selected. Then the concentration-time curves of these voxels were averaged, smoothed and truncated to avoid the second pass of the tracer.

### MRI Analysis

Acute ischemic lesions in the basal ganglia, thalamus, internal capsule, corona radiata or brainstem were classified as LI attributable to single perforating artery occlusion if the maximal diameter was < 15 mm on DWI. An underlying large vessel pathology was excluded on MR angiography. Ischemic lesion size was measured on DWI by manually delineated region of interest (ROI), summation of these areas in cm^2^ on each section and multiplication by the interslice spacing, to determine the volume in cm^3^ by use of OsiriX, a multidimensional image navigation and display software [20]. Lesion growth as well as reversal were defined as the difference between the infarction volume on follow-up DWI images and the initial DWI lesion volume.

To obtain information about hemodynamic alterations caused by vascular pathology, calculated TTP images demonstrating the delay of the contrast agent arrival in the brain parenchyma were used for visual analysis. Time to peak maps were classified as showing a normal perfusion pattern or areas of hypoperfusion. We defined mismatch as a hypoperfused area on PWI larger than the DWI lesion (PWI>DWI), inverse mismatch as a hypoperfused area smaller than the DWI lesion (PWI<DWI), and match when hypoperfusion and DWI lesion were equal (PWI=DWI).

In addition, the perfusion maps were quantitatively assessed by use of SPIN: a region of interest covering the hypoperfused area was placed on the generated maps (CBF, CBV) and mirrored to the contralateral unaffected hemisphere. Finally, ratios between the physiological estimates (CBF, CBV) of the lesion and of the contralateral mirror ROI were determined.

### Statistical analysis

All statistical analyses were performed using Statistical Product and Service Solutions (SPSS) statistics for Windows (Release 17.0; SPSS, Chicago, IL, USA). Comparison of lesion size on DWI and PWI was performed using the student's t-test. Comparison of CBF and CBV ratios as well as hypoperfused brain tissue volumes was performed using the student's t-test. Association of DWI reversal or lesion growth and hypoperfusion as well as treatment with intravenous thrombolysis was analyzed using Fisher's exact test. All statistics was performed with a 0.05 level of significance.

## Results

### Baseline characteristics and clinical presentation

In the final analysis 16 patients were included. The median age was 67 years (range 43-84 years); 14 (87.5%) patients were male, 2 (12.5%) patients were female. Table 1 shows the baseline characteristics in each patient. Overall, 11 (68.8%) patients were admitted to our hospital within 4.5 hours and 5 (31.2%) within six hours after onset of symptoms. Clinical presentation comprised dysarthria (50%), hemiparesis (81.3%), and hemihypaesthesia (18.8%), whereas other symptoms like disorientation, gait disturbances or oculomotor dysfunction were observed only occasionally. With regard to typical vascular risk factors, arterial hypertension (68.8%), hyperlipidaemia (50%), history of smoking (37.5%), and diabetes mellitus (31.3%) were observed most frequently 

**Table 1:** Demographics, clinical symtpoms, acute treatment, MRI findings, and clinical course.

**No**	**Age**	**Sex**	**rtPA**	**Side**	**DWI Lesion**	**DWI Lesion Volume**	**PWI**	**CBF**	**CBV**	**DWI Lesion Volume**	**Clinical course**
						**(cm^3^)**		**(ratio)**	**(ratio)**	**(cm^3^), follow-up**	
1	45	M	yes	R	Internal capsule	0.35	n			0.58	improvement
2	53	M	yes	L	Thalamus	0.25	n			0.35	improvement
3	54	M	yes	L	Corona radiata	1.01	n			1.06	improvement
4	70	M	no	R	Mesencephalon	0.18	n			0.26	improvement
5	71	M	no	R	Thalamus	0.35	n			0.35	improvement
6	84	M	no	L	Thalamus	0.46	n			0.49	improvement
7	43	M	yes	L	Internal capsule	0.86	↓	1.03	1.18	0.30	improvement
8	58	M	no	L	Corona radiata	0.40	↓	0.67	1.00	0.77	improvement
9	58	M	no	L	Internal capsule	0.00	↓	0.38	0.35	0.47	improvement
10	64	M	yes	L	Corona radiata	0.91	↓	0.60	0.71	0.59	stable
11	65	F	no	L	Thalamus	0.15	↓	0.97	1.54	1.28	deterioration
12	69	M	no	L	Internal capsule	0.29	↓	1.00	1.08	0.63	improvement
13	71	F	no	R	Thalamus	0.51	↓	0.33	0.53	1.65	deterioration
14	79	M	yes	L	Thalamus	0.51	↓	0.74	1.22	0.00	improvement
15	80	M	no	R	Thalamus	0.39	↓	0.39	0.43	0.39	stable
16	84	M	yes	R	Thalamus	0.75	↓	0.40	0.40	1.50	improvement

Legend: F: female, M: male; L; left; R: right; n normal, ↓ decreased, CBF: cerebral blood flow, CBV: cerebral blood volume.

### Acute treatment and in-hospital clinical outcome

Intravenous thrombolysis with recombinant tissue plasminogen activator (rtPA) was performed in 7 (43.8%) patients. The remaining patients were not treated because of a minor clinical deficit (n=4) or exceeded time window (n=5). A stable clinical course was observed in 2 (12.5 %), a deterioration in 2 (12.5%) and an improvement in 12 (75%) patients.

### MRI analysis

**Figure 1:**
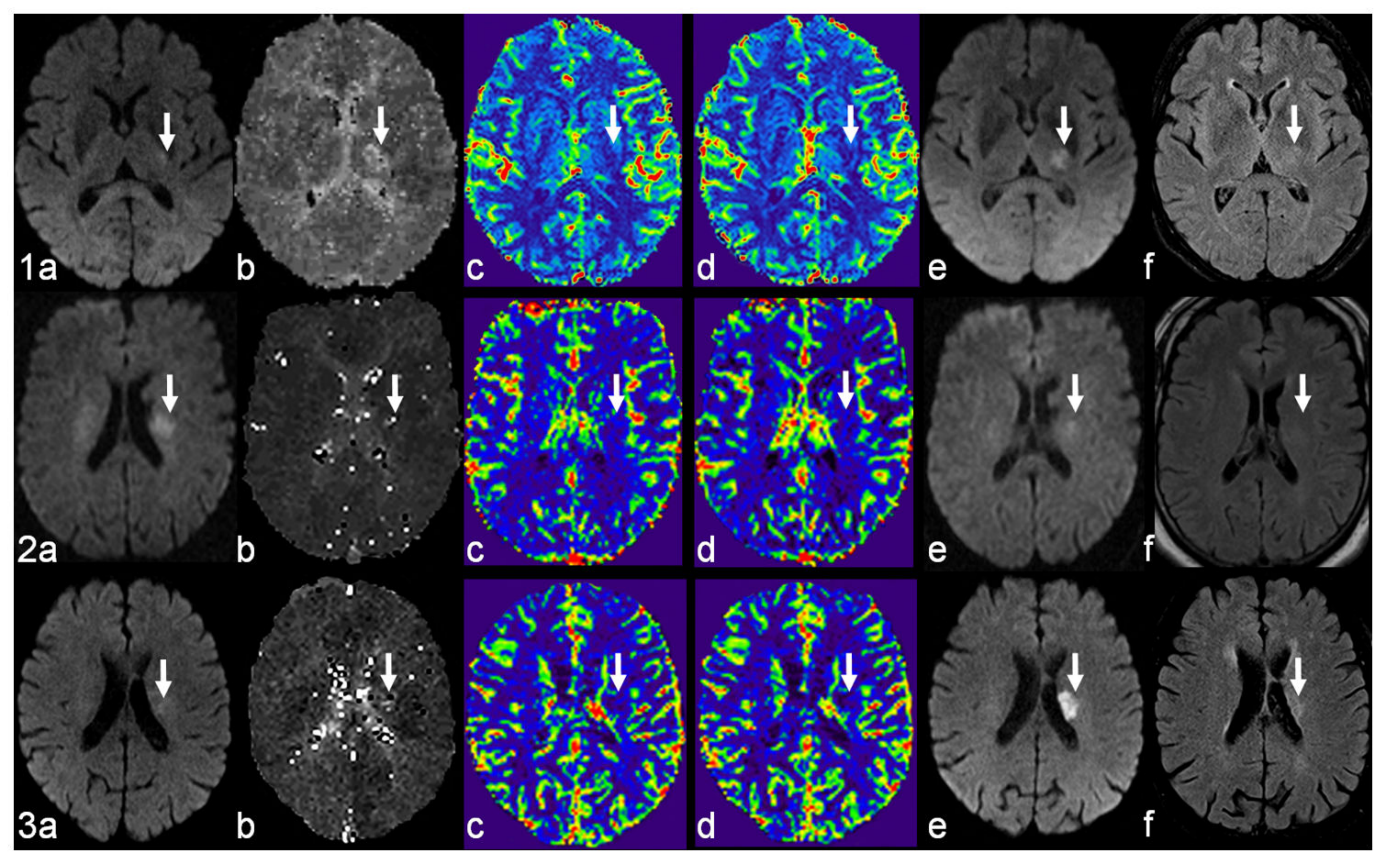
Three examples of perfusion patterns in acute lacunar infarction. Mismatch (1), inverse mismatch (2), and match (3) between diffusion-weighted and perfusion-weighted images (DWI, PWI). DWI (a) shows the initial ischemic lesion (white arrow). PWI derived maps demonstrate the perfusion deficit: time to peak (b), cerebral blood flow (c), and cerebral blood volume (d). Follow-up DWI (e) and FLAIR (f) show the ischemic lesion (white arrow). Note that case 2 is also an example of lesion reversal after intravenous thrombolysis.

Diffusion weighted imaging demonstrated an acute ischemic infarction in the thalamus in 8 (50%), in the internal capsule in 4 (25%), in the corona radiata in 3 (18.8%) patients, and in the mesencephalon in 1 (6.3%) patient. The right side was affected in 6 (37.5%), and the left side in 10 (62.5%) patients. On initial DWI, the ischemic lesions had a mean volume of 0.46±0.29 cm^3^. On follow-up DWI, the ischemic lesions had a mean volume of 0.67±0.47 cm^3^. Growth of DWI lesions was observed in 7 (43.8%) patients, reversal of DWI lesions in 3 (18.8%) patients (for details see Table 1 and Figure 1).

Perfusion weighted imaging showed a circumscribed hypoperfusion in 10 patients (62.5%), whereas the other perfusion studies were normal. The hypoperfused areas had a mean volume of 0.61±0.48 cm^3^. In comparison to the corresponding DWI lesion size (0.47±0.30 cm^3^) there was no significant difference (p=0.50). The mean CBF and CBV ratios were 0.65±0.28 and 0.84±0.41 respectively. With regard to the predefined perfusion patterns, 3 patients demonstrated a match, 4 an inverse mismatch, and 3 a mismatch. Figure 1 shows exemplary cases of each perfusion pattern.

Reversal of the DWI lesion was observed only in patients with hypoperfusion on PWI irrespective of the perfusion pattern (3/10 vs. 0/6, p=0.25) and treated with intravenous thrombolysis (3/7 vs. 0/9, p=0.63). In detail there were 2 patients with match and 1 patient with inverse mismatch. Lesion growth was shown in 6 patients with hypoperfusion but only in one patient with unremarkable perfusion study (6/10 vs. 1/6, p=0.15) and was more common in patients who did not receive intravenous thrombolysis (5/9 vs. 2/7, p=0.36). In detail there were 3 patients with mismatch, 2 patients with inverse mismatch and 1 patient with match. 

No significant differences in CBF (0.79±0.22 vs. 0.59±0.29, p=0.33) and CBV ratios (1.04±0.28 vs. 0.76±0.45, p=0.37) as well as hypoperfused brain tissue volumes (0.60±0.24 cm^3^ vs. 0.61±0.57, p=0.97) in patients with DWI lesion reversal were observed compared to those patients with stable or growing ischemic lesion size.

## Discussion

The most striking result of the present study of DWI-PWI patterns in LI is that all patterns that are well-known in large territorial infarction (match, inverse mismatch, mismatch) can also be observed in patients with LI. In particular the finding of a DWI-PWI mismatch in three LI patients was unexpected as comparable cases are not described in the medical literature. Due to a minor clinical deficit all three patients were not treated with intravenous thrombolysis and two of them showed an early clinical deterioration which was paralleled by a marked infarction growth on follow-up MRI. Whether the different DWI-PWI patterns represent different stages in stroke evolution or various underlying etiologies, however, remains open and needs to be addressed in further studies. Another important finding is the lesion reversal in three patients with LI after intravenous thrombolysis. Thus, acute therapy with rtPA may be regarded as an effective treatment option in this stroke subtype.

Although the existence of a penumbra has been demonstrated in an animal model of LI[21], until today no reports on its widely used MRI surrogate, the DWI-PWI mismatch have been published. In general, the medical literature concerning combined DWI and PWI in LI is sparse and the few reported study results are controversial. In one of the first studies with 6 patients, the authors observed no perfusion deficit at all in LI[6]. In contrast to this, Chalela and colleagues reported on a single patient with LI on DWI and matching hypoperfusion on PWI successfully treated with intravenous thrombolysis. Treatment was followed by a clinical improvement and reversal of the initially observed DWI abnormalities on follow-up MRI[7]. Similarly, we observed a partial or complete reversal of lesions in 3 patients. In the same year, another small case series (n=6 patients) demonstrated perfusion abnormalities to be present in all patients, but in all cases the perfusion deficits were found to be smaller than the initial ADC lesion. In the course, an increase in size of ADC as well as PWI abnormalities were found. Perfusion deficits always remained smaller than the corresponding ADC lesion. In the present series, however, only 4 of 10 patients with perfusion deficit demonstrated an inverse mismatch. The authors hypothesized that these lesions spread beyond the hypoperfused area possibly induced by cytotoxic mechanisms at the border of the tissue damage[8]. The results were confirmed in a larger case series featuring 19 patients more recently by the same study group[13]. In thalamic infarction, a DWI-PWI mismatch was only found in stroke due to large vessel disease or cardioembolism but not in LI in 5 patients[9]. In another study on DWI and PWI in acute ischemic infarction, all patients with LI (n=4) had a perfusion deficit matching the DWI abnormalities[10]. In the only larger case series, hypoperfusion on PWI was not only observed in two thirds of LI (n=22) but could also be related to an early clinical deterioration. Hypoperfusion was not associated with infarction growth, nor with a less favorable clinical outcome at 3-month follow-up. This stands in contrast with our own observation of infarction growth in 7 patients with LI and perfusion deficit. However, the authors did not attempt to determine the presence of a DWI-PWI mismatch[11]. In the brainstem, a matching hypoperfusion is far less common in lacunar pontine infarction(n=5) than in pontine infarction due to large vessel disease[12].

Similar to these inconsistent and partly contradictory results, the question whether intravenous thrombolysis is beneficial in patients with LI still has not been answered convincingly. Several authors are of the opinion that intravenous thrombolysis might be effective only in large vessel occlusion[15] and more recently proposed an observational study in order to define more precisely the effectiveness of rtPA in acute ischemic infarction without large arterial occlusion[16]. This hypothesis may be supported by the finding that three fourths of ischemic stroke patients without angiographically detectable vessel occlusion in the acute phase finally had a favorable clinical outcome at 3-month follow-up[22]. Pathophysiological considerations may also challenge the clinical practice to treat LI patients with intravenous thrombolysis. In the 1960ies and 70ies Fisher demonstrated in his meticulous pathoanatomical studies that LI are caused by occlusion of small perforating arteries due to lipohyalinosis or microatheroma[2]. Thus, rtPA might be ineffective to recanalize the occluded vessel in LI since it is an enzyme activating fibrinolysis. Furthermore, as pointed out earlier some authors also hypothesize a different pathophysiological mechanism in LI compared to large vessel occlusion: Infarction growth may not be the consequence of hypoperfusion but instead of cytotoxic mechanisms at the border of the initial ischemic lesion[8,13].

However, given the low mortality of LI, most of autopsies took place months to years after the index event and as a consequence, the observed changes may represent an already organized vascular lesion[23]. In the end, the pathoanatomical appearance of the vascular lesion and the underlying pathophysiological processes in acute LI remain unknown and the efficacy of rtPA cannot be excluded a priori.

The only available data from a large clinical trial derives from the National Institute of Neurological Disorders and Stroke study (NINDS) in which 81 patients with LI were included. Patients who received rtPA were more likely to have a favorable clinical outcome in comparison to patients who received the placebo[24]. Furthermore, several case series and larger observational studies addressed the important question of rtPA safety and effectiveness in acute LI. In a case series, Lee and colleagues found that hemorrhagic complications are unusual in patients with LI (n=13) treated with rtPA[25]. In a cohort study, patients with LI (n=101) treated with thrombolysis had the best clinical outcome of all stroke subtypes and did not suffer from symptomatic intracerebral hemorrhage[26]. Fuentes and colleagues did not observe a difference in 3-month clinical outcome between LI (n=60) and other stroke subtypes[27]. Correspondingly, in the largest cohort study published 2012 no difference in treatment response and a low rate of symptomatic intracerebral hemorrhage were observed in LI (n=195) compared to other stroke subtypes[28]. Compared to the reported frequency of LI (up to 25%), the rates of thrombolysed LI patients are rather low, ranging from 4 to 11%. There are several possible explanations for the reluctance to treat patients with LI. First, LI is still thought be associated with a benign prognosis in general[29]. Second, clinical presentation can be rather mild and deterioration may occur only later in the clinical course[4,30]. Finally, pathophysiological considerations might also restrain stroke neurologists from treating acute LI with intravenous thrombolysis. With regard to this ongoing debate, the findings of our study may support the argumentation in favor of an acute treatment in patients with LI[16].

The present study has some limitations. First, this is a retrospective study of moderate size since only patients who underwent initial MRI within 6 hours after symptom onset and follow-up MRI within one week were included. Follow-up MRI was primarily performed in patients who underwent intravenous thrombolysis or demonstrated clinical deterioration and this may explain the high thrombolysis rate of 43.1% in this study. Second, although all included patients underwent follow-up MRI, only 4 patients had follow-up PWI. Thus, we cannot draw any conclusions about the evolution of perfusion abnormalities with time. Third, characterization of penumbra in LI with multimodal MRI is difficult due to the small lesions, the limited spatial resolution of PWI and its susceptibility to motion artifacts. As a consequence punctuate hypoperfusion might be missed in small lacunar lesions. While the manual definition of the ROIs for measurement of the perfusion deficits may be considered to be a drawback of this study, a similar approach was used in other earlier studies [6,12]. Although we cannot exclude an under- or overestimation of PWI lesion size by this approach, there are no generally accepted or recommended PWI quantitative analysis approaches in LI. Even automated delineation of PWI lesions may result in exaggerated volumes as recently reported by Galinovic and colleagues [31]. Fourth, the diagnosis of LI is based on the clinical syndrome, characteristic MRI findings, and the exclusion of competing etiologies like cardioembolism or large vessel disease. Since the underlying vascular lesion could only be determined by neuropathological examination, some uncertainty remains regarding the final diagnosis. Finally, a detailed comparison of patients treated with intravenous thrombolysis and those without acute treatment was not performed due to the moderate study size. Consequently, the results have to be interpreted cautiously and should not be regarded as proof of efficacy of thrombolysis in acute LI.

In conclusion, multimodal MRI including DWI and PWI is feasible to demonstrate a mismatch in acute LI as well as growth or reversal of an ischemic lesion and consequently may serve for a better characterization of this stroke subtype and support treatment decisions in daily clinical practice.
